# Left ventricular hypertrophy and diastolic function in children and adolescents with essential hypertension

**DOI:** 10.1186/s40885-015-0031-8

**Published:** 2015-10-22

**Authors:** Heirim Lee, Young-Hwa Kong, Kyung-Hee Kim, June Huh, I-Seok Kang, Jinyoung Song

**Affiliations:** Department of Pediatrics, Samsung Medical Center, Sungkyunkwan University School of Medicine, 81 Irwon-ro, Gangnam-gu, Seoul 135-710 South Korea

**Keywords:** Left ventricular hypertrophy, Diastolic dysfunction, Pediatric essential hypertension, Ambulatory blood pressure monitoring

## Abstract

**Introduction:**

Left ventricular hypertrophy and diastolic dysfunction in children and adolescents with essential hypertension tend to be underdiagnosed. The aims of this study were to investigate left ventricular hypertrophy and diastolic dysfunction in the subjects with essential hypertension defined by ambulatory blood pressure monitoring.

**Methods:**

A total of 38 Korean subjects aged 9–19 years without secondary causes of hypertension were reviewed. Ambulatory blood pressure monitoring was done in the 38 subjects to diagnose hypertension and gain the information of blood pressure pattern. The subjects were divided into two groups: a group with elevated blood pressure (BP) index (*n =* 29) and a group with normal BP index (*n =* 9). Two-dimensional ultrasound with M-mode imaging and tissue Doppler imaging were performed to measure left ventricular mass index and to assess the left ventricular diastolic dysfunction.

**Results:**

Left ventricular mass index(g/m^2.7^) was significantly higher in the group with elevated BP index than the group with normal BP index, but there were no differences in left ventricular diastolic dysfunction evaluated by E/A ratio and E/E’ ratio. Left ventricular mass index was related only with body mass index, while any of the ambulatory blood pressure monitoring parameters did not predict left ventricular hypertrophy. In terms of diastolic dysfunction in essential hypertension, E/E’ ratio in the subjects with left ventricular hypertrophy was higher than that in the other subjects without left ventricular hypertrophy.

**Discussion:**

Left ventricular mass index is significantly correlated with body mass index in children and adolescents with essential hypertension, and the diastolic dysfunction could be in higher risk in subjects with left ventricular hypertrophy.

## Introduction

Essential hypertension in children is a major health issue, as its prevalence in children and adolescents has risen in accordance with the rise in childhood obesity. The current prevalence of hypertension in children ranges from approximately 1 to 4.5 % in the USA and will continue to increase. Longitudinal studies have shown that children with hypertension are likely to become hypertensive adults with an elevated cardiovascular risk [1].

Left ventricular hypertrophy (LVH) and diastolic dysfunction is the most documented type of cardiac damage that occurs in children and adolescents with hypertension. However, a recent study showed that only a quarter of adolescents with essential hypertension had been examined with echocardiography [2]. As they are clinically silent, LVH and diastolic dysfunction tend to be underdiagnosed, but LVH is associated with cardiac disease and mortality in adults. Therefore, early detection of the risk factors for cardiovascular organ damage may improve future prognosis.

In this study, the diagnoses of hypertension were made using 24-h ambulatory blood pressure monitors (ABPM). ABPM is a favorable method in treating childhood hypertension to provide diurnal blood pressure (BP) patterns [3]. We intended to investigate the prevalence of LVH and diastolic dysfunction in the subjects diagnosed with essential hypertension. The secondary aim of this study was to analyze the correlation factors of LVH and diastolic dysfunction. With several anthropometric characteristics, ABPM parameters were investigated as potential correlation factors to evaluate the effect of sustained hypertension on cardiac damage.

## Methods

### Patients

Between 2010 and 2014, a total of 55 Korean subjects aged from 9 to 19 years were enrolled in this retrospective study. Subjects were diagnosed with hypertension at other hospitals and referred to Samsung Medical Center for further evaluation and treatment. Of 55 subjects, 17 subjects with secondary hypertension were excluded on the basis of medical history, clinical exams, and several investigations. The 38 remaining subjects were divided in two groups according to their ABPM results: subjects with elevated BP index (the essential hypertension group) (*n =* 29) and subjects with normal BP index (the control group) (*n =* 9). The study was approved by the institutional review board (IRB) of the Samsung Medical Center. Informed consent was waived by the IRB.

### Ambulatory blood pressure monitoring

All ABPM measurements were obtained on an outpatient basis using the same type of device (Tonoport V monitor, GE, Germany). An appropriate cuff chosen for the patient’s arm size was attached to the non-dominant arm. The ABPM device checked the patient’s BP at intervals of 15 to 30 min in their actual personal life. The patients recorded actual daily activities while they were monitored. The BP index was calculated as mean daytime BP divided by the 95th percentile for gender and height by referring to the normalized reference values from the German Working Group study [4]. The BP load was defined as a percentage of the BP readings above the 95th percentile during the daytime. Essential hypertension was defined as mean daytime systolic BP index ≥1.0. Night dipping was defined as the decrease of mean of nocturnal BP readings for 10 % of the mean of the daytime readings.

### Echocardiography

Echocardiographic examinations were performed using Vivid E9 (GE Healthcare). The same cardiologist performed echocardiography on all subjects to evaluate left ventricular structure and diastolic function. The subjects were in the resting state in the left lateral position. The left ventricular end-diastolic internal dimension (LVIDd), end-diastolic septal thickness (IVSd), and posterior wall thickness (PWTd) were measured at end-diastole by two-dimensional guided M-mode echocardiography. The left ventricular mass was calculated according to the equation LVM(g) = 0.8 {1.04(IVSd + LVIDd + PWTd)}^3^ − (LVID)^3^ + 0.06. The left ventricular mass index was calculated by dividing the LVM(g) by the height in meters^2.7^ to correct the left ventricular mass (LVM) for body size. The left ventricular hypertrophy was defined as left ventricular mass index (LVMI) >51 g/m^2.7^ [5].

The left ventricular diastolic function was assessed by classic pulsed-wave Doppler technique. The mitral inflow velocities were obtained in the apical four-chamber view. The measurements of early diastolic peak flow velocity (E), E-wave deceleration time (DT), late diastolic peak flow velocity (A), and E/A ratio were determined. In this study, the left ventricular diastolic dysfunction was defined as E/E’ ratio >8 or E/A ratio <1.0.

### Data analysis

Data were expressed as mean ± SD or proportion (%) of patients. SPSS 21.0 was used for the statistical analysis. For the evaluation of the difference between two independent groups, the nonparametric Mann-Whitney *U* test and Fisher’s exact test were used.

The Spearman correlation coefficient and logistic regression test were used to predict the left ventricular hypertrophy and diastolic dysfunction. The statistical significance was defined when *p* < 0.05.

## Results

### Essential hypertension group vs control group

The anthropometric characteristics, blood pressure characteristics, and echocardiographic findings of 29 hypertensive subjects and nine controls are described in Table 1. Age was not different between the two groups. The office BP values of the essential hypertension group were measured as hypertensive definitely [6], while four of nine subjects (44.4 %) with normal BP index turned to be truly normotensive. As expected, the hypertensive subjects had a significantly higher BP index (*p* < 0.00) and BP load (*p* < 0.001) than those in the control group. However, there was no statistical difference in night dip between the two groups. Compared to those in the control group, the subjects with hypertension had a significantly higher BMI (*p* = 0.001). LVMI (g/m^2.7^) was significantly higher in the essential hypertensive group, but LVH(%) was not higher in the controls. No difference was found in Tei index and LV diastolic function evaluated by E/A ratio and E/E’ ratio between the two groups.

**Table 1:** Normal BP index (BP index <1.0) vs high BP index (BP index ≥1.0)

	BP index <1.0	BP index ≥1.0	*p* value
*N*	9	29	
Age (year)	15.0 ± 2.6	15.7 ± 2.0	0.624
BMI	20.2 ± 2.7	26.3 ± 5.3	0.001
BP index	0.94 ± 0.06	1.11 ± 0.09	<0.001
BP load (day) (%)	19.6 ± 17.9	81.2 ± 20.5	<0.001
Night dip (%)	7.0 ± 5.4	8.5 ± 4.2	0.164
Daytime average SBP	125.9 ± 7.4	151.1 ± 11.6	<0.001
Night time average SBP	117.0 ± 7.7	139.9 ± 13.9	<0.001
LVMI (g/m^2.7^)	32.2 ± 8.1	40.7 ± 9.8	0.016
LVMI (g/m^2^)	73.2 ± 17.6	84.2 ± 16.8	0.032
LVH (*N*) (%)	0 (0 %)	5 (19.2)	0.297
E/E’	7.6 ± 2.5	7.1 ± 2.2	0.583
E/A	1.9 ± 0.5	1.9 ± 0.7	0.854
Tei index	0.3 ± 0.1	0.3 ± 0.1	0.929

Test method = Mann-Whitney *U* test

*N* sampler number, *BMI* body mass index, *BP index* blood pressure index, *BP load (day)* blood pressure load during daytime, *LVMI* left ventricular mass index, *LVH* left ventricular hypertrophy, *E/E’* early transmitral filling velocity/early diastolic tissue velocity at septal mitral annulus, *E/A* early transmitral filling velocity/late transmitral filling velocity

### Left ventricular hypertrophy and LV diastolic dysfunction in essential hypertension

LVH was detected in four of 29 subjects with essential hypertension (13.8 %). The correlation analysis showed that there was a significant quantitative linear relationship between LVMI and BMI (Spearman rho CC = 0.676, *p* < 0.001) (Table 2). However, the left ventricular mass index did not correlate with any of the ABPM parameters (Table 3). Logistic regression analysis was performed to investigate the risk factors of LVH in hypertensive subjects (Table 4). BMI was the only significant risk factor of LVH. Age and the ABPM parameters did not predict LVH.

**Table 2:** Correlation analysis of LVH in essential hypertension

		Age (year)	BMI	BP index	BP load	Night dip	Day SBP	Night SBP	E/E’	Tei index
LVMI (g/m^2.7^)	Correlation coefficient	0.050	0.676	0.301	0.096	0.171	0.186	0.239	0.165	−0.120
	*p* value	0.807	0.000	0.136	0.640	0.405	0.334	0.212	0.432	0.568

Test method = Spearman rho correlations

*Day SBP* daytime average systolic blood pressure, *Night SBP* night time average systolic blood pressure

**Table 3:** Risk factor analysis for LVH

	B	S.E.	Wals	DOF	*p* value	Exp(B)	95 % confidence interval	
							Lower	Upper
Age	−0.299	0.328	0.833	1	0.362	0.742	0.390	1.409
BMI	0.522	0.261	4.013	1	0.045	1.685	1.011	2.809
BP index	−11.457	13.785	0.691	1	0.406	0.000	0.000	5730413.639
BP load	0.011	0.035	0.094	1	0.759	1.011	0.944	1.083
Night dip	0.250	0.155	2.589	1	0.108	1.284	0.947	1.740

Test method = logistic regression analysis

**Table 4:** Risk factor analysis for LV diastolic dysfunction

	B	S.E.	Wals	DOF	*p* value	Exp(B)	95 % confidence interval	
							Lower	Upper
BMI	0.333	0.210	2.501	1	0.114	1.395	0.932	2.107
BP index	−8.128	9.440	0.741	1	0.389	0.000	0.000	32003.376
BP load	−0.027	0.030	0.769	1	0.381	0.974	0.917	1.034
LVMI	−0.69	0.086	0.642	1	0.423	0.934	0.790	1.104
Night dip	−0.006	0.114	0.003	1	0.959	0.994	0.795	1.243

Test method = logistic regression analysis

**Fig. 1:**
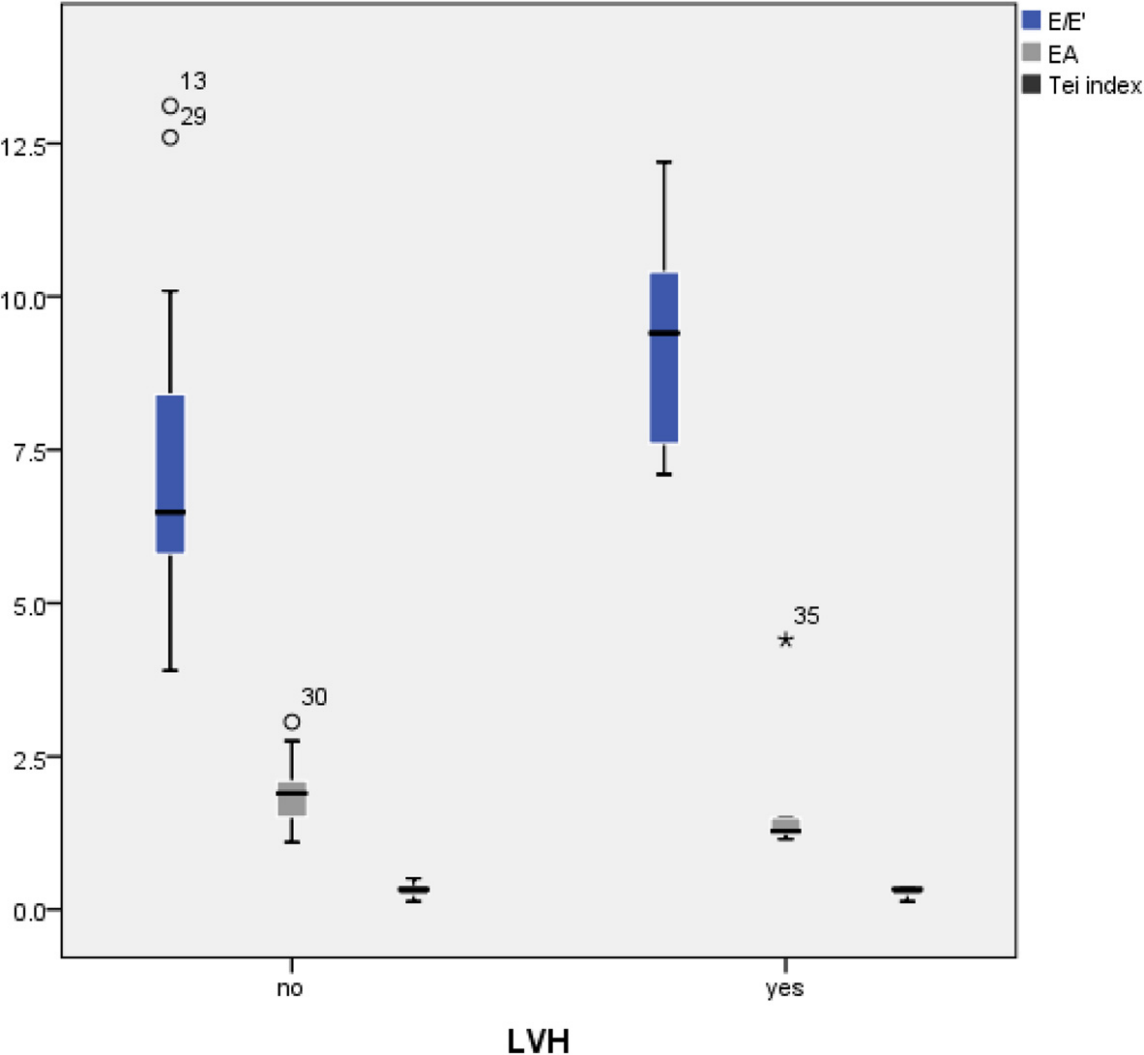
Differences of LV diastolic dysfunction in the presence of LVH. E/E’ ratio of hypertensive subjects was significantly higher in the group with LVH. On the other hand, there was no difference of E/A ratio and Tei index according to the presence of LVH or not

LV diastolic dysfunction was found in seven of 28 hypertensive subjects (25 %). The risk factor analysis indicated that the ABPM parameters, anthropometric measurements, and LVMI did not predict LV diastolic dysfunction. We investigated the relationship between LVH and LV diastolic function by comparing the hypertensive subjects with LVH with those without LVH. Even though no differences in E/A ratio and Tei index were found between the two groups, only E/E’ was elevated in the group with LVH compared to those without LVH (*p* = 0.014) (Fig. 1) (Table 5).

**Table 5:** Differences of LV diastolic dysfunction in the presence of LVH

	LVH (−)	LVH (+)	*p*
E/E’	6.8 ± 2.0	9.3 ± 2.1	0.014
E/A	1.9 ± 0.5	1.9 ± 1.4	0.174
Tei index	0.3 ± 0.1	0.3 ± 0.1	0.54

Test method = *t* test

## Discussion

In this study, we analyzed LVH and diastolic function in children and adolescents with essential hypertension. We compared the essential hypertension group to the group with normal BP index. Strictly speaking, the control group in our study is different from normal control. The control group contains subjects with white coat hypertension (WCH) as well as normal subjects. A previous study showed that patients with WCH have increased cardiovascular risk in the aspect of target organ damage compared with a normotensive group [7]. However, subjects with essential hypertension are more likely to be exposed to hypertension than subject with WCH. Hence, our results are consistent with the prevailing view that both hypertension and obesity are independent risk factors of LVMI. LVM is commonly indexed by the body surface area to normalize mass for the body size of pediatric patients. However, in this study, LVM was indexed by height^2.7^ in order to avoid errors in estimating the impact of overweight. Because the LVMI indexed by the BSA considers obesity as a physiological variable, it does not allow identification of significant differences between BMI subgroups [5, 8].

In the literature, the risk factor identified for LVH in hypertensive subjects was BMI. Many studies have been published concerning the development of LVH in obese children and adolescents. The pathophysiological mechanism of obesity-related LVH has been well described. In obese patients, increased metabolic requirements lead to an increase in preload and afterload to the heart and an adaptive increase in LVM to normalize the increased wall tension [9]. Obesity is not related only to eccentric hypertrophy of the heart, but also to diastolic dysfunction. A recent study indicated that the echocardiographic parameters of diastolic function (E/E’ and pulmonary vascular flow velocity) were independently associated with obesity in normotensive adolescents. In severely obese subjects, diastolic dysfunction was significantly related to the presence of high cardiometabolic factors, such as apolipoprotein A1, soluble vascular cell endothelial molecules-1 (sVCAM-1), and retinol-binding protein 4 (RBP4) [10]. Therefore, in obese pediatric patients, thorough management of BMI and body adiposity is important to minimize irreversible changes in the cardiovascular system and persistent cardiovascular risk factors.

Diastolic dysfunction is a well-described type of cardiovascular organ damage in pediatric patients with hypertension. Diastolic dysfunction progresses with myocardial fibrosis and ischemia in chronic systemic hypertension. Therefore, LV diastolic dysfunction with restriction in the left ventricular filling is considered a hallmark of hypertensive heart disease [11]. Several studies have shown that there are statistically significant parameters indicating LV diastolic dysfunction has been observed in children with primary arterial hypertension with the use of tissue Doppler imaging (TDI) [12]. In adults, TDI has been used as a predictor for cardiovascular risk monitoring of raised left ventricular diastolic pressure [13]. In this study, it failed to show a correlation of essential hypertension with LV diastolic dysfunction. Differences in sample size and the severity of obesity could explain the observed differences.

ABPM is considered superior to standard BP measurement in the evaluation of target organ damage related to hypertension [3]. Usually, the diurnal change of BP is related to target organ damage. Under normal circumstances, the mean of nocturnal BP readings is at least 10 % lower than that of daytime BP readings. The lack of a nocturnal fall in BP and higher nocturnal BP values suggest the presence of target organ damage in primary hypertension [3].

In this study, ABPM parameters, such as BP load and night dip, had no significant correlation with LVH or diastolic function. On the other hand, other studies suggested that the ABPM data of hypertensive and prehypertensive adolescents were associated with pathologically elevated LVMIs. They confirmed that LVMI is higher in sustained hypertensive adolescents than in normotensives, but the LVMI of white coat hypertensives did not differ [14]. A recent study suggested that the rise in BP in the morning is an important factor of cardiac damage that influences the development of abnormal relaxation [15]. Another study showed that peak systolic blood pressure on the exercise test was the only independent predictor of LVMI, although its overall contribution was relatively low [16]. Despite the conflicting view, our results suggest that it is hard to evaluate target organ damage with ABPM parameters alone and there are many potential correlation factors to be investigated.

In this study, we defined essential hypertension as mean daytime systolic BP index ≥1.0. Several studies showed that daytime and 24-h average BP may indeed carry similar information for diagnosing hypertension in clinical practice [17, 18]. Furthermore, night time BP measured by ABPM device may not always reflect night dip accurately because many patients are disturbed in their sleep with ABPM devices. A previous study showed that sleep disturbance during overnight BP monitoring increases the nocturnal BP level and potentially attenuates the correlation with hypertension-related cardiac damage [19]. Because of the small study population, erroneous measurements of nocturnal BP are likely to be resulted in this study. Hence, we used the mean day time systolic BP index as our primary indices of essential hypertension.

Hypertension is one of the most important modifiable risk factors in the development of cardiac diseases. In children, both physiological and pathological increases in blood pressure progressively modify the geometry of the left ventricle, causing a significant increase in its wall thicknesses. As essential hypertension is usually asymptomatic, cardiovascular damage is usually clinically silent in the early stages. However, cardiac mass is already subject to change during the early hypertensive stages [1]. Hence, early echocardiographic screening in hypertensive adolescents before the progression of cardiac damage is needed to improve future cardiovascular health.

Potential limitations of the current study include the retrospective study design and a relatively small sample size. We did not collect all the echocardiographic data of all the subjects. Nevertheless, the fact remains that the results in this study are statistically significant. We did not compare essential hypertensive group to normal controls entirely. The retrospective study design precluded the evaluation of the subjects’ current BP status and cardiac function. These results are hardly applicable to different geographic populations. In addition, the LVMI and diastolic function were assessed by echocardiography and pulse-wave Doppler echocardiography, which is a less objective method than other precise techniques, such as cardiac MRI. Nonetheless, the imaging techniques we used are commonly used in clinical practice to detect LVH and LV diastolic dysfunction successfully.

## Conclusion

In conclusion, LVMI significantly correlated with BMI in children and adolescents with essential hypertension based on ABPM. However, the ABPM parameters of sustained hypertension did not predict left ventricular hypertrophy. LVH was related to LV diastolic dysfunction, as indicated by the elevated E/E’ ratio in children and adolescents with essential hypertension.

## Abbreviations

A: late diastolic peak flow velocity
ABPM: 24-h ambulatory blood pressure monitors
BMI: body mass index
BP: blood pressure
DT: E-wave deceleration time
E: early diastolic peak flow velocity
E’: early diastolic tissue velocity at septal mitral annulus
IVSd: end-diastolic septal thickness
LVH: left ventricular hypertrophy
LVID: left ventricular internal dimension
LVIDd: left ventricular end-diastolic internal dimension
LVM: left ventricular mass
LVMI: left ventricular mass index
PWTd: end-diastolic posterior wall thickness
RBP4: retinol-binding protein 4
SD: standard deviation
sVCAM-1: soluble vascular cell endothelial molecules-1
TDI: tissue Doppler imaging
WCH: white coat hypertension
